# Calibrating COVID-19 susceptible-exposed-infected-removed models with time-varying effective contact rates

**DOI:** 10.1098/rsta.2021.0120

**Published:** 2022-01-10

**Authors:** James P. Gleeson, Thomas Brendan Murphy, Joseph D. O’Brien, Nial Friel, Norma Bargary, David J. P. O'Sullivan

**Affiliations:** ^1^ MACSI, Department of Mathematics and Statistics, University of Limerick, Limerick, V94 T9PX, Ireland; ^2^ School of Mathematics and Statistics, University College Dublin, Dublin, D04 V1W8, Ireland; ^3^ Insight Centre for Data Analytics, Ireland; ^4^ Confirm Centre for Smart Manufacturing, Ireland; ^5^ Irish Epidemiological Modelling Advisory Group (IEMAG), Ireland

**Keywords:** epidemic modelling, differential equations, calibration, generalized additive model, thin-plate splines

## Abstract

We describe the population-based susceptible-exposed-infected-removed (SEIR) model developed by the Irish Epidemiological Modelling Advisory Group (IEMAG), which advises the Irish government on COVID-19 responses. The model assumes a time-varying effective contact rate (equivalently, a time-varying reproduction number) to model the effect of non-pharmaceutical interventions. A crucial technical challenge in applying such models is their accurate calibration to observed data, e.g. to the daily number of confirmed new cases, as the history of the disease strongly affects predictions of future scenarios. We demonstrate an approach based on inversion of the SEIR equations in conjunction with statistical modelling and spline-fitting of the data to produce a robust methodology for calibration of a wide class of models of this type.

This article is part of the theme issue ‘Data science approaches to infectious disease surveillance’.

## 1. Introduction

The Irish Epidemiological Modelling Advisory Group (IEMAG) was established in March 2020 to provide expert advice to Ireland’s Chief Medical Officer and National Public Health Emergency Team on COVID-19 responses. As part of a suite of mathematical and statistical modelling tools, we developed a population-level susceptible-exposed-infected-removed (SEIR) model based on multiple compartments [1–3]. Many groups have used such models to aid in scenario-based planning for pandemic responses, e.g. [4–6]. Although population-level SEIR models use a number of simplifying assumptions such as a fully mixed and homogeneous population—a simplification that is avoided by more complex age-cohorted or agent-based models [7–11], for example—they enable rapid analysis of potential policy interventions and can give clear quantification of the uncertainty due to the limited knowledge of virus parameters. Calibration of such models to the observed data—in our case, the number of daily cases of COVID-19 in Ireland—is an important technical challenge that is heightened by the noisy nature of the data and the uncertainty in parameter estimation. In this article, we describe the SEIR model used by IEMAG and give a detailed description of the calibration algorithm, an early version of that appeared in the technical report [12]. We emphasise the uncertainty quantification that is enabled by this approach, and we highlight the adaptability of the calibration framework, which enables it to be applied—under certain conditions that we examine in detail—to other models that may be required for future pandemics.

The calibration of SEIR models to noisy data has attracted much attention both before and during the COVID-19 pandemic. Our basic assumption is that the effective contact rate β of the model can be considered as time-varying to model the impact of non-pharmaceutical interventions such as working from home, closure of schools and universities, lockdown, etc.; the challenge lies in estimating this time-varying β(t). Bayesian and inverse-problem methods for parameter estimation are well established [13,14], but these usually assume that all parameters are constant in time or allow only piecewise-constant variations in β, with changes in β at a predefined set of breakpoints (e.g. the dates that movement restrictions are changed). In contrast, we follow the direction of Mummert [15], who showed that a time-varying effective contact rate can be found for susceptible-infected-removed (SIR) systems by an exact inversion of the governing differential equations of the model. In extending this concept, we generalize to a range of models and derive conditions on the model structure and on the smoothness of the data-fitting function, which are required for this approach to be successful. The smoothness conditions are satisfied by statistical models for data fitting, of which we focus here on the negative binomial generalized additive model (GAM). We note that Goswami *et al.* [16] have applied similar ideas to invert SEIR models for COVID-19 data, but they use the raw (unfitted) data and therefore occasionally obtain negative estimates for β(t). They can obtain a smoothed (and non-negative) form by a polynomial fit to the recovered β values; in contrast, we use a smooth fit to the data and do not require any post-processing of the β(t) values.

The remainder of this article is structured as follows. In §2, we introduce the SEIR model and discuss methods for its numerical solution. Section 3 presents our main results on calibration, including the GAM model and the algorithm (and conditions) for determining the time-varying effective contact rate. In §4, we give examples of the application of the algorithm and model to investigate scenarios and to demonstrate its applicability to more complex models, such as SEIR models that include vaccination. We draw conclusions in §5; details of calculations and model properties are included in the electronic supplementary material file.

## 2. The Irish Epidemiological Modelling Advisory Group susceptible-exposed-infected-removed model

Population-level SEIR models [1,2] assume fully mixed, homogeneous populations. Despite this simplification, they provide useful information for scenario-based planning, with the potential for further extension of the structure (e.g. to dis-aggregated age cohorts) in more advanced models.

### (a) Model structure

At each moment of time, every individual in the population is considered to be in one of a discrete number of compartments. The structure of the compartments, and the timescales for individuals to move in and out of compartments, are based on the current understanding of the epidemiology of COVID-19, as evidenced by the extensive literature review and evidence synthesis conducted by [17–19].

**Figure 1.**
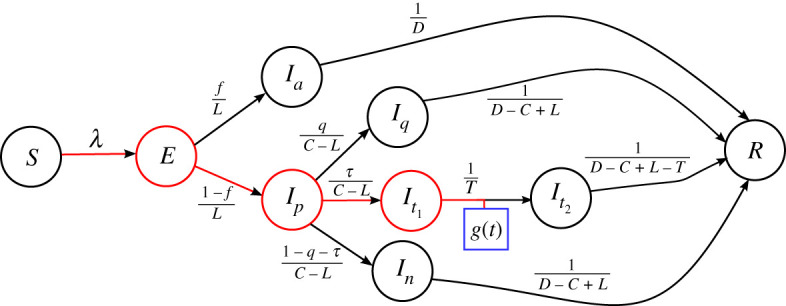
Representation of the model of equations (2.1)–(2.11) as a weighted, directed network or graph. Each node (vertex) represents a compartment of the model, and each edge (link) shows the direction of flow of individuals. The out-edges from a node are weighted by the probability of exiting the node along this edge divided by the average residence time in the compartment of that node. The blue square marks the source of the observed data, fitted by g(t) (see §3(a)), which is the daily number of newly reported confirmed cases. This is related to the flux from ${\text{I}}_{t_{1}}$ to $I_{t_{2}}$ by equation (2.11). The red edges and red nodes are discussed in Conditions 3.1 and 3.2 of §3(b).

The compartments (S, E, etc.) of the model, see figure 1, are labelled by the state of the individuals who are assumed to flow through the model structure as their infection progresses. The corresponding mathematical variables (S(t), E(t), etc.) represent the number of individuals—from a homogeneous population of fixed size N—in each of the respective compartments at time t. Those individuals who are in the susceptible (S) compartment can, should they share a contact with an infected individual that enables transmission of the virus, become exposed (enter the E compartment). While individuals are in the exposed compartment (for an average of L days), the virus is still latent so they do not display symptoms, nor are they infectious.

At the end of their latent period, we assume that a fraction f of exposed individuals enter the infected-asymptomatic (${\text{I}}_{a}$) compartment, where they do not develop symptoms, but they may infect others (with a probability of infection that is a factor h<1 lower than that of symptomatic infected individuals). The remaining fraction 1−f of individuals exiting the exposed compartment flow into the pre-symptomatic-infected (${\text{I}}_{p}$) compartment. They remain in the ${\text{I}}_{p}$ compartment for an average duration of C−L days, where C is the incubation period of the virus, and while there they do not show symptoms but they are infectious. Symptoms are assumed to develop at the exit time from the ${\text{I}}_{p}$ compartment, and there are three routes that individuals may take: with probability q they self-isolate and quarantine while infectious (in the ${\text{I}}_{q}$ compartment), with probability τ they undergo a COVID-19 test and isolate while awaiting their result (in the ${\text{I}}_{t_{1}}$ compartment) and the remaining cases (probability 1−q−τ) are assumed to not quarantine and remain in the community while infectious (${\text{I}}_{n}$ compartment). In all cases, the average period of infectiousness is denoted by D, while the duration in the pre-symptomatic compartment is C−L so that, for example, the average time spent in the ${\text{I}}_{q}$ or ${\text{I}}_{n}$ compartments is D−(C−L).

Those individuals who are symptomatic and tested flow from the ${\text{I}}_{{\text{t}}_{1}}$ compartment to the ${\text{I}}_{t_{2}}$ compartment when their test result is confirmed: this occurs an average time T after their symptoms appeared (so the average residence time in the ${\text{I}}_{t_{1}}$ compartment is T and that in the ${\text{I}}_{{\text{t}}_{2}}$ compartment is D−(C−L)−T). The main output of the model is the number of new confirmed cases per day, which is the flux from the ${\text{I}}_{t_{1}}$ compartment to the ${\text{I}}_{t_{2}}$ compartment. After an average duration D of infectiousness, all individuals either recover or are removed (hospitalized or die) and are accounted for in the R compartment.

The flows described earlier are expressed in terms of differential equations for the time-dependent variables S(t), E(t), etc. as follows:

2.1 $$\begin{array}{r} \frac{\text{d}S}{\text{d}t}=-\lambda S \end{array}$$

2.2 $$\begin{array}{r} \frac{\text{d}E}{\text{d}t}=\lambda S-\frac{1}{L}E \end{array}$$

2.3 $$\begin{array}{r} \frac{\text{d}I_{a}}{\text{d}t}=\frac{f}{L}E-\frac{1}{D}I_{a} \end{array}$$

2.4 $$\begin{array}{r} \frac{\text{d}I_{p}}{\text{d}t}=\frac{\;(1-f)}{L}E-\frac{1}{C-L}I_{p} \end{array}$$

2.5 $$\begin{array}{r} \frac{\text{d}I_{q}}{\text{d}t}=\frac{q}{C-L}I_{p}-\frac{1}{D-C+L}I_{q} \end{array}$$

2.6 $$\begin{array}{r} \frac{\text{d}I_{t_{1}}}{\text{d}t}=\frac{\tau }{C-L}I_{p}-\frac{1}{T}I_{t_{1}} \end{array}$$

2.7 $$\begin{array}{r} \frac{\text{d}I_{t_{2}}}{\text{d}t}=\frac{1}{T}I_{t_{1}}-\frac{1}{D-C+L-T}I_{t_{2}} \end{array}$$

2.8 $$\begin{array}{r} \frac{\text{d}I_{n}}{\text{d}t}=\frac{\left(1-q-\tau \right)}{C-L}I_{p}-\frac{1}{D-C+L}I_{n} \end{array}$$

2.9 $$\begin{array}{r} \;\text{and}\;\frac{\text{d}R}{\text{d}t}=\frac{1}{D}I_{a}+\frac{1}{D-C+L}I_{q}+\frac{1}{D-C+L-T}I_{t_{2}}+\frac{1}{D-C+L}I_{n}, \end{array}$$

where S(t) is the number of susceptible individuals, E(t) is the number who are exposed, $I_{p}(t)$ is the number who are pre-symptomatic infected, $I_{a}(t)$ is the number who are asymptomatic infected, $I_{q}(t)$ is the number who are symptomatic and self-isolating (without testing), $I_{t_{1}}(t)$ is the number who are symptomatic and waiting for testing, $I_{t_{2}}(t)$ is the number who are in post-test self-isolation, $I_{n}(t)$ is the number who are symptomatic and not isolating and R(t) is the number who are removed. Time t is counted in days from 28 February 2020, to match the timing of the first confirmed cases of COVID-19 in Ireland.

The force of infection λ(t) that appears in equations (2.1) and (2.2) is the time-dependent rate at which susceptible individuals acquire the disease [2]. This is given by the effective contact rate β(t) (the total contact rate multiplied by the risk of infection given contact between an infectious and a susceptible person) multiplied by the probability that a contact (in a well-mixed population) is effectively infectious, given by the weighted sum over infectious compartments divided by population size N, so λ(t) can be written as follows:

2.10 $$\lambda (t)=\beta (I_{p}+hI_{a}+iI_{q}+I_{t_{1}}+jI_{t_{2}}+I_{n})/N.$$

Here, the parameters h, i and j are multiplicative factors to model the reduction of effective transmission from, respectively, the asymptomatic infected (${\text{I}}_{a}$), symptomatic quarantining (${\text{I}}_{q}$) and post-test isolation (${\text{I}}_{t_{2}}$) compartments, relative to symptomatic infected.

In addition, we define $C_{c}(t)$ to be the cumulative number of new cases reported by time t, given by integrating the flux out of the ${\text{I}}_{t_{1}}$ (waiting-for-test) compartment:

2.11 $$\frac{\text{d}C_{c}}{\text{d}t}=\frac{1}{T}I_{t_{1}},$$

and we also report the number of new daily cases on day t, defined by $c_{c}(t)=C_{c}(t)-C_{c}(t-1)$.

The ranges assumed for all parameters are guided by literature reviews [17–19] and are summarized in the electronic supplementary material.

### (b) Finite-difference formulation

The system of differential equations (2.1)–(2.11) may be solved numerically using, for example, a simple forward-Euler scheme. This scheme approximates the derivative dx/dt by $(x_{n+1}-x_{n})/\Delta$, where Δ is the finite timestep and $x_{n}$ is the discrete-time approximation to x(n Δ), the value of x(t) at time t=n Δ. Expressing the dynamical system (2.1)–(2.11) in the general form

2.12 $$\frac{\text{d}x}{\text{d}t}=F(x(t)),$$

where x(t) represents the vector of all unknowns, and using the finite-difference approximation enables the solution $x_{n}$ to be determined from the initial condition $x_{0}$ by iteration:

2.13 $$x_{n+1}=x_{n}+\Delta \;F(x_{n})\;\text{for}\;n=0,1,2,\ldots .$$

To ensure the accuracy of this finite-difference formulation, it is important that the timestep Δ chosen be sufficiently small: the convergence of the finite-difference solution to the differential equation solution occurs in the limit Δ→0. Testing of the finite-difference results for the system (2.1)–(2.11) shows that a value of Δ equal to 0.1 days gives sufficient accuracy.

### (c) Network representation

It proves convenient to represent the model structure described earlier as a directed weighted graph or network, see figure 1. The nodes (vertices) of the graph represent the compartments of the model, while each directed edge shows the direction of flow of individuals as the disease progresses. The out-edges from a node are weighted by the probability of exiting the node along this edge divided by the average residence time in the compartment of that node. Consider, for example, the two out-edges from the E node in figure 1. The edge leading to the asymptomatic infected compartment (node ${\text{I}}_{a}$) has weight f/L because a fraction f of individuals flow along the path, while the average time spent in the E compartment is L. Similarly, the edge leading to the pre-symptomatic compartment (node ${\text{I}}_{p}$) has weight (1−f)/L. The output of the model is the flux (number of individuals per unit time) along the edge from ${\text{I}}_{{\text{t}}_{1}}$ to ${\text{I}}_{{\text{t}}_{2}}$, and this is marked as g(t) in figure 1. We shall show in §3 that this directed graph structure facilitates extensions of the model to include more complicated structural features.

Representing the network by its weighted (and time-dependent) adjacency matrix, with $a_{ij}(t)$ being the weight of the directed edge from node i to node j at time t (and $a_{ij}=0$ if there is no edge from node i to node j), note that the system of differential equations (2.1)–(2.11) is succinctly expressed as follows:

2.14 $$\frac{\text{d}x_{j}}{\text{d}t}=\sum_{i}a_{ij}x_{i}-\sum_{k}a_{jk}x_{j},$$

where $x_{j}(t)$ is the number of individuals in compartment j at time t. In this equation, the first term on the right-hand side sums over the in-edges to node j, while the second term is the sum of the outflows from node j. Although this appears to have the form of a linear system, note that the force of infection λ(t)—which depends on the infectious compartments—is an element of the adjacency matrix, which is therefore time dependent, and so the system is nonlinear. Nevertheless, the form of equation (2.14) can be exploited to enable calibration of the model to data.

## 3. Calibration

As COVID-19 spread, governments of most countries enacted non-pharmaceutical interventions to slow the growth in the number of cases. These interventions typically aim to reduce the effective contact rate β so that there are fewer opportunities for the virus to be transmitted from an infectious to a susceptible person. We therefore assume that the effective contact rate parameter β in equation (2.10) is time dependent, and we seek to determine what this rate should be in order to reproduce the observed data on the number of confirmed cases. The process we describe here is similar in principle to the method described in [15] (and references therein) for the SIR model, but complicated by the additional compartments of this SEIR model.

### (a) Data and generalized additive model

The data of interest are the confirmed numbers $c_{c}(t)$ of COVID-19 cases per day in Ireland. The confirmed positive case data were extracted from the Computerised Infectious Disease Reporting (CIDR) database hosted by the Health Protection Surveillance Centre. The event dates of the cases were used to calibrate the model, with the daily case counts $c_{c}(t)$ at day t being modelled by a negative binomial random variable

3.1 $$c_{c}(t)∼NegB(g(t),\theta ),$$

where g(t) is the expected number of cases on day t and θ is the overdispersion parameter. The negative binomial distribution is a natural distribution to model the variation in $c_{c}(t)$ around the mean function g(t). This is because empirically the variance of $c_{c}(t)$ is observed typically to be greater than the mean g(t), in other words, an over-dispersed distribution is appropriate. In particular, an alternative such as the equi-dispersed Poisson distribution would give rise to a poorer fitting model for $c_{c}(t)$. Under the negative binomial model $\text{E}[c_{c}(t)]=g(t)$ and $\text{Var}[c_{c}(t)]=g(t)+\theta g{\left(t\right)}^{2}$, which allows us to interpret θ as an overdispersion parameter. In particular, the negative binomial converges to the equi-dispersed Poisson distribution in the limit θ→0.

To model the mean parameter g(t) of the negative binomial distribution, we use a thin-plate regression spline [20],

3.2 $$\log g(t)=\beta_{0}+\sum_{k=1}^{K}\beta_{k}B_{k}(t),$$

where $\left(\beta_{0},\beta_{1},\ldots ,\beta_{K}\right)$ are unknown parameters and $\left\{B_{k}(t):k=1,2,\ldots ,K\right\}$ are thin-plate spline basis functions; the value of K is chosen to be large enough to achieve a satisfactory goodness of fit. The resulting model is a negative binomial GAM [21,22].

To account for parameter uncertainty, equations (3.1) and (3.2) were fitted in a Bayesian framework using the brms R package [23–25]. The prior distributions for the model parameters are the defaults within the brms software: $\beta_{0}∼t_{3}(5.9,2.5)$, $\beta_{1},\ldots ,\beta_{K}∼t_{3}(0,2.5)$ and θ∼gamma (0.01,0.01). These are vague priors to reflect little *a priori* information about the parameter values. The brms R package interfaces with Stan [26] to generate samples from the posterior distribution for the model parameters; the posterior distribution is found to be relatively insensitive to the priors.

### (b) Inversion algorithm

The challenge of inverting the SEIR differential equations is the following: for a given set of model parameters and a given fit g(t) of the historical case data, to determine a time-varying effective contact rate β(t) and a set of initial conditions for the model of equations (2.1)–(2.11) so that the model output, equation (2.11), exactly matches to the fitted data of equation (3.2).

Although the steps outlined later can be performed analytically (see electronic supplementary material) with a view towards extensions of the model, it is helpful to consider instead the finite-difference approximation of the graph representation given by equation (2.14). Writing $x_{j,m}$ as the approximation for $x_{j}(m\Delta )$, the number of individuals in compartment j at timestep m, the finite-difference version of equation (2.14) can be rearranged to give

3.3 $$x_{j,m+1}=x_{j,m}+\Delta \left(\sum_{i}a_{ij,m}x_{i,m}-\sum_{k}a_{jk,m}x_{j,m}\right)\;\text{for}\;m=0,1,\ldots ,$$

where we write $a_{ij,m}$ to denote the (i,j) entry of the time-dependent adjacency matrix at timestep m. Note that if the time dependence of all compartments that are in-neighbours of compartment j is known (i.e. if $x_{i,m}$ is known for all m for those compartments i that have $a_{ij,m}>0$), and if an initial condition $x_{j,0}$ is given for compartment j, then equation (3.3) provides an explicit iterative scheme to determine $x_{j,m}$ for all m. This means that if the time dependence is known for all in-neighbour nodes of node j, then the value of $x_{j}$ can be fully determined.

A partial converse result also exists. Suppose that the time dependence of compartment j is known for all time (i.e. $x_{j,m}$ is known for all m) and also assume that node j only has one in-neighbour, denoted node i. Then, the first sum on the right-hand side of equation (2.14) reduces to a single term, and the corresponding finite-difference approximation can be rearranged to yield

3.4 $$x_{i,m}=\frac{1}{a_{ij,m}}\left(\frac{x_{j,m+1}-x_{j,m}}{\Delta }+\sum_{k}a_{jk,m}x_{j,m}\right)\;\text{for}\;m=0,1,\ldots .$$

Thus, the time dependence of nodes who have a single ‘parent’ (in-neighbour) node can, if known, be used to determine the time dependence of the parent node, including the initial condition $x_{i,0}$ of the parent node.

These two properties—the ability to determine time dependence of nodes from the time dependence of all in-neighbours, equation (3.3), and the partial converse of determining the time dependence of a single-parent node from that of its ‘child’ node, equation (3.4)—form the basis of the algorithm that allows calibration of the model to output data from a range of graph-represented models.

The first step of the algorithm is to link the model’s time-dependent output directly to the time dependence of a compartment (node). In all our models, the output can be expressed in terms of the cumulative number of confirmed cases $C_{c}(t)$ and if this is given (e.g. by the GAM fit of equation (3.2)) so that $\text{d}C_{c}/\text{d}t=g(t)$ is a known function, then equation (2.11) can be inverted to determine ${\text{I}}_{t_{1}}(t)$ as follows:

3.5 $${\text{I}}_{t_{1}}=Tg(t).$$

Considering the graph representation in figure 1, we note that node ${\text{I}}_{{\text{t}}_{1}}$ has a single in-neighbour or parent node, ${\text{I}}_{p}$, and so equation (3.4) can be employed to determine the time dependence of compartment ${\text{I}}_{p}$ from the known (from equation (3.5)) time dependence of ${\text{I}}_{t_{1}}$. Similarly, ${\text{I}}_{p}$ has a single-parent node, E, whose time dependence can be determined from another application of equation (3.4). These steps of the algorithm—from a node to its single parent—are marked with red edges in figure 1 and these link the time dependence of nodes ${\text{I}}_{t_{1}}$, ${\text{I}}_{p}$ and E directly to the data-fitting function g(t).

Recalling from equation (2.10) that the force of infection λ(t) is a nonlinear function of the infectious compartments, some of which have (as yet) unknown time dependence, the calculation for S(t) is slightly more involved. It proves convenient to define an auxiliary variable ω(t) as ω(t)=λ(t)S(t), so that equation (2.2) can be rearranged to give

3.6 $$\omega =\frac{\text{d}E}{\text{d}t}+\frac{1}{L}E.$$

Since we have already determined the time dependence of E(t), at least through its finite-difference approximation, this relationship gives us the time dependence of the auxiliary variable ω(t). Then, noting that equation (2.1) is dS/dt=−ω, we can determine the time dependence of the susceptible compartment from its initial disease-free condition S(0)=N by integration of ω(t) or, in the finite-difference approximation, by summation.

We also need to determine the time dependence of those infectious compartments that are not already marked as red in figure 1. The tree-like structure of the infectious nodes, with the E compartment as the ‘root’ of the tree, means that the time dependence of all the nodes can be determined by repeated application of equation (3.3) along with the assumption that the initial condition for any of the ‘children’ nodes is zero. In this way, the known time dependence of variable E(t) determines $I_{a}(t)$, while knowing $I_{p}(t)$ allows for $I_{q}(t)$ and $I_{n}(t)$ to be determined. Finally, $I_{t_{2}}(t)$ can be determined from ${\text{I}}_{t_{1}}$ in the same way.

At this stage in the algorithm, we have determined the time dependence of all infectious compartments and of the auxiliary variable ω(t). We can therefore rearrange equation (2.1) to explicitly solve for the effective contact rate β(t) as

3.7 $$\begin{array}{rl} \beta (t) & =-\frac{N}{S}\frac{\text{d}S}{\text{d}t}{\left(I_{p}+hI_{a}+iI_{q}+I_{t_{1}}+jI_{t_{2}}+I_{n}\right)}^{-1} \end{array}$$

3.8 $$\begin{array}{rl} & =\frac{N\omega }{S}{\left(I_{p}+hI_{a}+iI_{q}+I_{t_{1}}+jI_{t_{2}}+I_{n}\right)}^{-1}. \end{array}$$

Although all the steps outlined earlier can also be performed analytically by solving algebraic and linear differential equations (see electronic supplementary material), the finite-difference formulation is particularly useful when considering the generalization of the method to other models that are represented by larger graphs. Therefore, it is worth pausing here to consider the three crucial aspects of the graph structure that are exploited in the algorithm. We express these as three conditions for the method to be applicable. In order to do so, we define the concepts of ‘red path’ and ‘red nodes’, reflecting the colours used for edges and nodes, respectively, in figure 1.

**Condition 3.1.**

A reverse-direction ‘red path’ exists from the edge of the data-fitting function g(t) to the force-of-infection edge (the edge from S to E that is weighted by λ), with each node on this red path having exactly one in-neighbour.

The links that constitute the red path for the IEMAG model are coloured red in figure 1. Next, we iteratively define the set of ‘red nodes’, by beginning with all nodes that are on the red path (these nodes are coloured red in figure 1). Then any node whose in-neighbours are all red nodes is also labelled as a red node. We continue this iteration until no further nodes become red nodes, and then the following condition must hold for the calibration algorithm to apply.

**Condition 3.2.**

All infectious-compartment nodes are red nodes.

A third condition is that the data-fitting function g(t) must be sufficiently smooth. We show in the electronic supplementary material that the following condition is necessary:

**Condition 3.3.**

The function g(t) must have at least as many derivatives as the number of edges in the red path.

Condition 3.1 enables the identification of the time dependence of the red-path nodes from the data by the application of equation (3.4), while Condition 3.2 subsequently allows all infectious compartments to be calculated (assuming zero initial condition) from the time dependence of the red-path nodes using equation (3.3). Once these time dependencies are calculated, the finite-difference values for the auxiliary variable ω(t)=λ(t)S(t) and subsequently for the effective contact rate β(t) are evaluated similar to equation (3.8). Condition 3.3 is required because each step on the red path from ${\text{I}}_{{\text{t}}_{1}}$ back to S involves differentiating the ‘child’ variable to obtain the ‘parent’ (in-neighbour) variable, so g(t) must be sufficiently smooth to allow this to occur for all steps on the red path. This has implications for extending the model to allow for compartmental residence time distributions that are Erlang rather than exponential, see electronic supplementary material.

## 4. Examples

**Figure 2.**
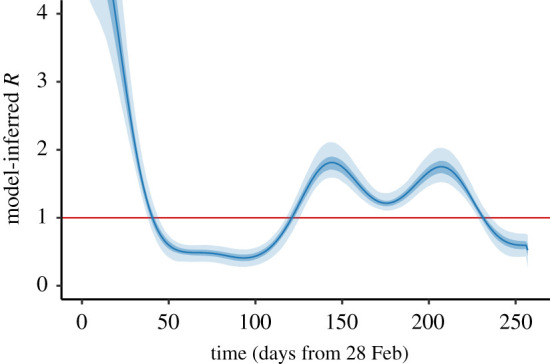
Model-inferred R up to 11 November 2020 (day 257 of the Irish epidemic), as described in §4(a). The curve indicates the mean (over 1000 realizations as described in §4); shaded regions show the 50% (quantiles 0.25–0.75) and 95% (quantiles 0.025–0.975) credible intervals. In this and subsequent figures, time is measured in days from 28 February 2020.

**Figure 3.**
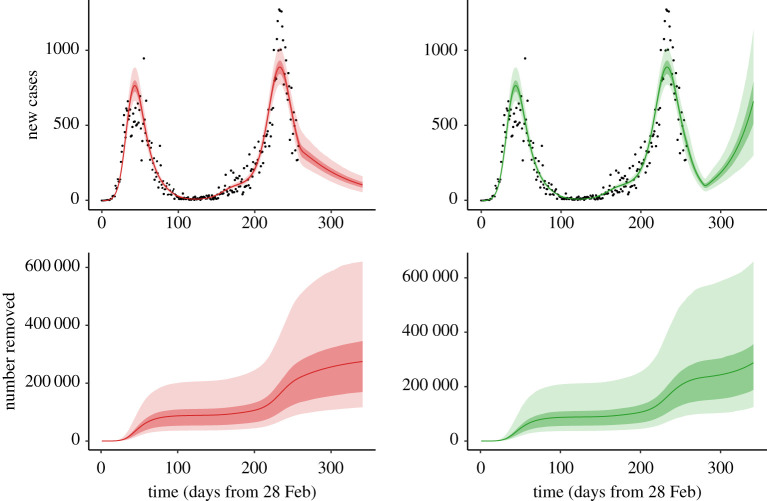
Scenario 1 (left panels) assumes R=0.9 from 11 November (day 257); scenario 2 (right panels) assumes R=0.5 from 11 November until 2 December (day 278), with R=1.4 thereafter. The top panels show confirmed new cases per day, with black symbols for the observed data; the bottom panels show the number of removed individuals (this includes both deaths and those who recovered from the virus). In each case, the curve indicates the mean (over 1000 realizations as described in §4); shaded regions show the 50% (quantiles 0.25–0.75) and 95% (quantiles 0.025–0.975) credible intervals.

To demonstrate the calibration algorithm of §3(b) and illustrate its application to scenario analysis, we use the confirmed number of COVID-19 cases per day in Ireland from 28 February 2020 to 11 November 2020, as described in §3(a). The role of IEMAG was to advise on policy decisions, for which various future scenarios were modelled. Given the evolving information on the virus parameters and the noisy nature of daily case data, it is important to quantify the uncertainty associated with any scenario prediction. Accordingly, for each of figures 2 and 3, a sample of 1000 posterior realizations of the curves are generated from the GAM fitting procedure (§3(a)), and for each such realization, independent draws from the distributions of the model parameters (see electronic supplementary material) are used within the calibration algorithm to generate scenario outputs.

### (a) Model-inferred reproduction number

The calibration algorithm described in §3 yields the time-dependent effective contact rate β(t) that is consistent with the GAM fit g(t) to the case data and with the set of model parameters selected in that realization. It is useful to communicate the values of β(t) in terms of a quantity that is closely related to a (time-varying) reproduction number. The basic reproduction number $R_{0}$ for compartmental models of the type studied here is known to be directly proportional to the effective contact rate β through the eigenvalue of a matrix (see electronic supplementary material). This allows us to express effective contact rates β in terms of ‘equivalent’ $R_{0}$ values, although care must be taken with interpreting such values as $R_{0}$ values are only well defined at the beginning of an epidemic. We call these ‘model-inferred R’ values. Using this relationship, the time-varying β(t) can be similarly expressed as a time-varying R value, and the β values considered in the scenario analyses later are described in terms of their equivalent model-inferred R values.

Figure 2 shows the model-inferred R (which is directly proportional to β(t)) determined by the calibration algorithm on 11 November 2020 (day 257), as a function of time in terms of days from 28 February 2020. The periods where R is below one correspond to declining case numbers in the population. While the times where R changes from above to below one, and vice versa, can be related to the days when population-level movement restrictions changed, they are not exactly the same, which demonstrates the utility of a data-driven approach to estimation rather than specifying breakpoints, where β(t) is assumed to change value.

### (b) Scenarios

For each set of model parameters, the calibration algorithm detailed in §3 yields a time-varying contact rate β(t) and a set of initial conditions that are consistent with the GAM-fitted data up to the calibration date of 11 November 2020. Forward predictions of the model from that date may then be examined under assumptions about how the contact rate (or, equivalently, the model-inferred reproduction number) may behave in the future.

As an example, in Scenario 1, shown in the left panels of figure 3, we assume that the effective contact rate will remain at a level equivalent to R=0.9 from the calibration date. Solving the finite-difference approximation (equation 2.13) of the differential equation system given by equations (2.1)–(2.11) yields the expected number of daily cases and other outputs, such as the number removed (number of individuals in the R compartment) under this assumption, see figure 3.

Scenario 2, in the right panels of figure 3, investigates an alternative possibility for the post-calibration-date contact rates. Here, the inferred reproduction number R is assumed to be 0.5 from 11 November (day 257) until 2 December (day 278), with R increased to 1.4 thereafter. It is important to note that in each case it is assumed that the reproduction number is exactly as specified in the scenario description. Comparing the results for the two scenarios, it is clear that the effect of uncertainty in future R values dominates the uncertainty due to the range of possible values for the other parameters of the model.

### (c) Incorporating vaccination into the model

As population-level vaccination is an important instrument for virus suppression, it is important to extend the model described in §2 to include the impact of policy decisions related to vaccine rollout.

**Figure 4.**
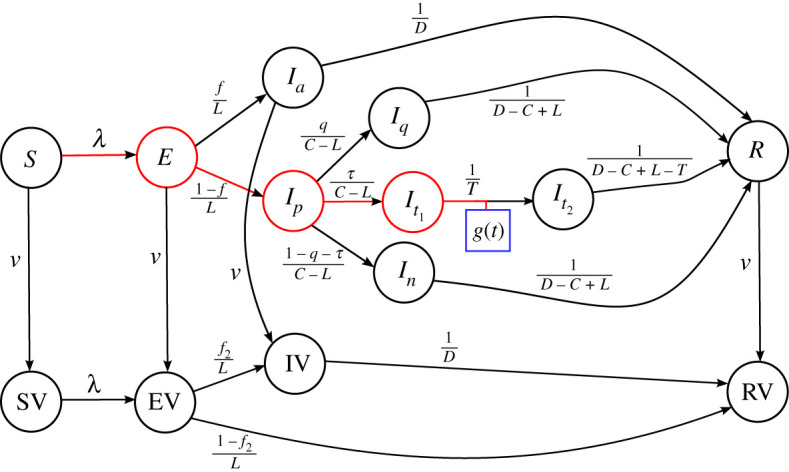
Extension of figure 1 to include compartments for effectively vaccinated individuals (SV, EV, IV and RV). The rate that individuals move from an unvaccinated compartment (e.g. *S*) to its effectively vaccinated analogue (e.g. SV) is given in terms of the number of vaccines administered daily by Eq. (S13) in the electronic supplementary material. The parameter $f_{2}$ is the fractions of individuals who, having been effectively vaccinated and exposed to the virus, become asymptomatically infected, see electronic supplementary material for further details.

**Figure 5.**
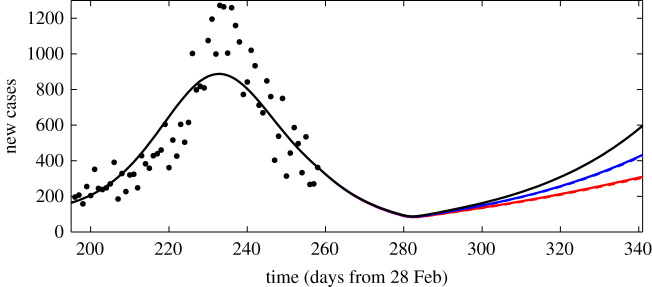
Daily new cases for a single realization of parameter values (see electronic supplementary material), assuming R=0.5 from 11 November (day 257) until 2 December (day 278), with R=1.4 thereafter. The black curve is from the model of equations (2.1)–(2.11), with no vaccinations. The blue and red solid curves are for scenarios from the vaccination model described in electronic supplementary material, which assume vaccines are administered at a rate of $v_{d}=5000$ per day (blue) or $v_{d}=10\;000$ per day (red), with the vaccines first becoming effective on 11 November. Almost indistinguishable from the solid curves are the blue and red dashed curves, which show the results of the reduced-order model described in electronic supplementary material for $v_{d}=5000$ and $v_{d}=10\;000$, respectively.

In the electronic supplementary material we describe a model that includes new compartments called SV, EV, IV and RV to contain those individuals who are effectively vaccinated while also being susceptible, exposed, (asymptomatic) infected or removed, respectively: see figure 4 for the network structure of the model. Figure 5 shows how vaccinations, assumed to be administered at a constant rate of $v_{d}$ per day, could impact on Scenario 2. For clarity, we show results only for one set of parameters (see electronic supplementary material for parameter values), but we illustrate the impact of the vaccination rollout speed by comparing results for scenarios where we assume either $v_{d}=5000$ or $v_{d}=10\;000$ vaccines are administered each day, becoming effective from 11 November (day 257). The calibration and scenario match that of Scenario 2, with an effective contact rate equivalent to R=0.5 switching to R=1.4 on 2 December (day 278).

For further extensions of the model, for example including age-cohort compartments for both vaccinated and unvaccinated individuals, it proves convenient to derive a reduced-order approximation to the vaccination model. The derivation of this reduced model is described in the electronic supplementary material. The results of the reduced model are shown by the dashed curves in figure 5, and they are almost indistinguishable from the full model results (solid curves), demonstrating the accuracy of the reduced model.

## 5. Conclusion

In this article, we have outlined the population-level SEIR model of COVID-19 that is used by the IEMAG. Our main result is the calibration algorithm of §3 that is sufficiently general to allow for extensions to the model structure. Our analysis identified three conditions on the graph structure of the model and the smoothness of the data-fitting function that are necessary and sufficient for the inversion algorithm to be applicable. We gave examples in §4 of the use of the model for scenario analysis and uncertainty quantification; we also demonstrated the adaptability of the calibration algorithm by applying it to a model with vaccination. While vaccination requires a substantial increase in the number of model compartments, we showed that an approximate reduced-order model can also give good accuracy at lower complexity.

There are several directions in which this work could be advanced. Our negative binomial GAM fits (equation (3.1)), for example, assume a constant overdispersion parameter, but this could be generalized to allow for time-dependent overdispersion. Another possible extension would be to consider multiple data sources as required, for example, in an age-cohorted SEIR model where daily confirmed cases are recorded for various age categories or to include time series data on hospitalizations, deaths or multi-strain variants in addition to case counts. We hope that the work presented here will form a basis for such extensions and that our focus on network-representation calibration will facilitate adoption of the approach for other SEIR-type pandemic models.

## Data accessibility

R code that implements the calibration algorithm described in §3 and runs an example scenario is available for download from https://github.com/obrienjoey/ireland_covid_modelling.
